# Mass Transport Properties of the Rabbit Aortic Wall

**DOI:** 10.1371/journal.pone.0120363

**Published:** 2015-03-17

**Authors:** Emma L. Bailey, Eleni Bazigou, Piotr S. J. Sowinski, Peter D. Weinberg

**Affiliations:** Department of Bioengineering, Imperial College London, London, United Kingdom; Public Health Research Institute at RBHS, UNITED STATES

## Abstract

Uptake of circulating macromolecules by the arterial wall may be a critical step in atherogenesis. Here we investigate the age-related changes in patterns of uptake that occur in the rabbit. In immature aortas, uptake was elevated in a triangle downstream of branch ostia, a region prone to disease in immature rabbits and children. By 16-22 months, uptake was high lateral to ostia, as is lesion prevalence in mature rabbits and young adults. In older rabbits there was a more upstream pattern, similar to the disease distribution in older people. These variations were predominantly caused by the branches themselves, rather than reflecting larger patterns within which the branches happened to be situated (as may occur with patterns of haemodynamic wall shear stress). The narrow streaks of high uptake reported in some previous studies were shown to be *post mortem* artefacts. Finally, heparin (which interferes with the NO pathway) had no effect on the difference in uptake between regions upstream and downstream of branches in immature rabbits but reversed the difference in older rabbits, as does inhibiting NO synthesis directly. Nevertheless, examination of uptake all around the branch showed that changes occurred at both ages and that they were quite subtle, potentially explaining why inhibiting NO has only minor effects on lesion patterns in mature rabbits and contradicting the earlier conclusion that mechanotransduction pathways change with age. We suggest that recently-established changes in the patterns of haemodynamic forces themselves are more likely to account for the age-dependence of uptake patterns.

## Introduction

Atherosclerosis is characterized by focal accumulations of lipids, fibrous proteins and cells within the arterial wall. The lipid originates predominantly from circulating lipoproteins and its preferential deposition at well-defined sites may therefore reflect variation in lipoprotein uptake by the wall. Evidence for a spatial correlation between such transport and the development of atherosclerosis derives particularly from studies of arterial branch points. Wall uptake of plasma macromolecules and the prevalence of lesions are both greater downstream than upstream of branch ostia in the immature rabbit aorta; conversely, they are both greater upstream than downstream of ostia in the mature rabbit aorta [1]. Note that because there is a change in the pattern of both properties, the spatial correlation is only revealed when comparisons are made at the same age.

The study of variation in wall transport properties has been hindered by laborious or limited techniques. We recently presented a new method based on tile-scanning confocal microscopy that quantifies fluorescent tracer concentrations in 3D with high spatial resolution over large areas, and is rapid and convenient [2]. The method was evaluated in pilot studies of macromolecule uptake near the origins of intercostal arteries in the rabbit aorta and a tile scan of tracer uptake in the entire descending thoracic aorta [2].

The studies reported in the present paper employed three improvements to the technique: (i) improved identification of the luminal surface reduced the acquisition of out-of-focus glare, giving a much less uniform pattern of uptake and improving the correspondence with lesion distributions; (ii) the 3D capabilities of the method were more fully exploited by projecting 2D maps of tracer uptake on 3D reconstructions of the aortic surface and by extracting profiles of tracer concentration at different depths into the wall; and (iii) the quantitative potential of the method was further developed to obtain maps of mass transfer coefficients, which can be compared absolutely one to another and to previous data, unlike maps with arbitrary fluorescence intensity scales.

The improved method was used to investigate four related questions concerning patterns of wall mass transport:

First, we investigated the hypothesis that there are more transport patterns than demonstrated hitherto. There are two patterns of fatty streaks around branch mouths in mature human aortas—a lateral pattern is seen in young adults, and an upstream streak is seen at later ages [3]. The pilot data obtained in our first confocal mapping paper identified only a single mature pattern of transport [2]. In the present study we used a wider range of ages to determine whether other patterns exist.

Second, we investigated whether patterns of transport around branches are determined by the branches themselves or occur, like some features of periositial wall shear stress variation [4], because the branches sit within larger patterns. We examined maps of larger aortic regions to determine whether their transport patterns explained the patterns around branches within them. We also compared branches on the left and right side of aorta, which should have identical transport patterns if such patterns are determined solely by the branch.

Third, we investigated the streaks of high uptake described in some previous studies e.g. [5], which are thought to explain the fact that lipid deposition also occurs in the form of streaks. Based on earlier reports that *in situ* collapse of the aorta leads to endothelial damage [6], we hypothesised that streaks occur at lateral margins of the aorta as a result of *post mortem* tracer uptake after depressurization; we tested this hypothesis by examining uptake in animals in which arteries were fixed under terminal anaesthesia rather than after death, and in animals where tracer was administered just prior to death.

Finally, we investigated the effect of heparin on patterns of uptake. Herparin displaces proteins bound to heparan sulphate in the endothelial glycocalyx, a putative transducer of haemodynamic wall shear stress, and it reduces flow-dependent dilatation of arterioles and the bioavailability of nitric oxide (NO) [7]. In turn, inhibition of NO synthesis *in vitro* [8] and, to a lesser extent, *in vivo* [9] modifies the mature pattern of transport, uptake becoming greater downstream rather than upstream of the branch. We therefore hypothesized that heparin would similarly alter the mature pattern.

## Methods

### Animals

All animal experiments complied with the Animals (Scientific Procedures) Act 1986 and were approved by the Local Ethical Review Process Committee of Imperial College London (Home Office Licence PPL 70/7333). Male New Zealand White rabbits (HSDIF strain, Specific Pathogen Free, Harlan UK) aged 9 weeks (1.7–2.1 kg), 6 months (2.7–3.4 kg) or 16–22 months (3.8–4.1 kg) (n≥4 rabbits/group) plus 2 females aged >5 years (5.2–5.3 kg) were individually housed in pens at 18–22°C on a 12:12 h light:dark cycle and fed a standard laboratory diet (SDS) with tap water *ad libitum*.

### In vivo protocol

Rhodamine-labelled albumin (150 mg/kg), prepared and purified of free dye as previously described [2], was administered via the marginal ear vein to conscious rabbits and allowed to circulate for 10 minutes. Heparin (2000 units) was administered via the same route 8 minutes after the tracer, followed at 10 minutes by an overdose of sodium pentobarbitone (Euthatal, Rhone Merieux; 160 mg/kg). After a midline thoracotomy and laparotomy, a blood sample was withdrawn from the left ventricle of the heart. A retrograde cannula was tied into the aorta at the level of the diaphragm and used to flush the thoracic segment for 30 s with saline from a reservoir ~100 cm above the animal. Formaldehyde was then administered by the same route. (A concentration of 15% was used as it gives substantially better tracer retention than the conventional 4% [2].) The maximum time between death and fixative entering the aorta was 5 minutes. The aortic arch was clamped after approximately 1 minute. The aorta was excised after 30 minutes of *in situ* fixation.

To confirm the low level of autofluorescence in the tracer channel seen in previous studies, tracer was omitted in some experiments. To investigate the effect of *post-mortem* uptake, tracer was administered 1 minute rather than 10 minutes before euthanasia in some experiments; this interval allowed mixing of tracer with the blood whilst minimising uptake under pressure. In other experiments to assess *post-mortem* uptake, fixation was conducted through an anterograde cannula tied into the proximal descending aorta; in some of these animals aortic pressure was maintained until the moment the cannula was introduced by conducting the procedure under anaesthesia (approx. 35 mg/kg pentobarbitone, anaesthetic depth being assessed from the pedal withdrawal reflex) whereas in others the cannulation was conducted after euthanasia, as before. To investigate effects of heparin, it was administered 1 h before the tracer [7] rather than 8 minutes after, and at a three-fold higher dose than used for anticoagulation (6000 units, *iv*).

### Preparation of tissue and plasma gels for confocal microscopy

The proximal segment of the excised descending thoracic aorta was opened along its ventral midline, pinned flat to a silicon gel and postfixed in 15% formaldehyde overnight at 4°C. The tissue was then mounted luminal side up on a microscope slide using mowiol-glycerol mounting medium with anti-fade agent (N-propyl-galate, Fluka) and left to set overnight at room temperature. Blood samples were centrifuged for 10 minutes at 13,000x*g* to obtain plasma, which was diluted 1:50 in 12.5% gelatin; 5μl samples were placed on a microscope slide and fixed in 15% formaldehyde at 4°C overnight.

### Confocal microscopy

An area of aorta encompassing at least the 2^nd^-4^th^ pairs of intercostal artery branch ostia was scanned *en face* using an inverted Leica SP5 laser scanning confocal microscope with a computer-controlled motorised stage. (The 1^st^ “pair” was excluded as often there is only a single branch at that location). Tissue autofluorescence and fluorescence from rhodamine-albumin were excited at 458 and 561 nm and detected at 465–515 and 585–620 nm, respectively. A 20x 0.7NA glycerol immersion lens was combined with tile scanning to give high resolution and a large field of view. The voxel size was 6 x 6 x 1 μm in x, y and z, respectively (x = aortic axis, y = circumferential direction, z = depth into the wall); a total depth of ~200–300 μm was imaged.

### Quantification of tracer uptake and statistical analyses

Each confocal data file consisted of a 3D matrix of voxel intensity values. Using in-house software, these matrices were imported into MATLAB (The MathWorks Inc). A median filter was used to reduce noise in the data, and a flatfield correction was applied to remove spatial biases in sensitivity—each XY plane in the matrix was divided by a corresponding 2D scan of a uniformly fluorescent slide (Chroma). The luminal surface was located in each z-wise column of voxels using an intensity threshold obtained for the autofluorescence channel matrix by the method of Otsu [10]. To obtain 2D maps of uptake in the inner wall, intensities were summed over a depth of 10μm from the surface into the tissue in each column. (In our previous study [2], the flatfield correction was applied to these 2D maps rather than the 3D matrix, and the Otsu threshold was obtained by averaging individual thresholds calculated for depth-wise slices through the autofluorescence matrix rather than by directly computing it for the entire matrix). Arbitrary intensities were converted to mass transfer coefficients (V, cm/s) using the equation V = (I_T_ d) / (I_P_ t), where I_T_ = intensity of fluorescence in tissue, I_P_ = intensity of fluorescence in plasma, d = distance into the wall (cm) and t = duration of tracer circulation (s). I_P_ was obtained by applying the Matlab analysis to the matrix obtained for the plasma gel, which was imaged using the same gain settings employed for the tissue.

The 2D maps were analysed further to determine patterns of mass transfer. Mass transfer coefficients were averaged within squares centred on intercostal branch ostia that were 1800 x 1800 μm in area for young animals and 2700 x 2700 μm for mature animals, the linear dimension corresponding to approximately three times the diameter of the branch at each age. Within each of these squares, mass transfer coefficients were also averaged in regions of interest (ROIs) upstream, downstream and at the sides of intercostal ostia. The ROIs had dimension of 450 x 600 μm for young animals and were appropriately scaled for mature animals—see Supporting Information S1 Fig. for an example. Variation of mass transfer coefficients in the circumferential direction was analysed in each map of a whole aortic segment by averaging coefficients in more elongated ROIs that were placed close to the left lateral margin and the right lateral margin of the map, between left branch ostia and between right branch ostia, and along the dorsal midline. These ROIs avoided the areas immediately around the branches themselves, and the narrow longitudinal streaks of high uptake described below.

### Statistics

Data were analysed by nested ANOVA, or ANOVA applied to regression in groups when it was necessary to account for the use of several branches from each rabbit, with p<0.05 as the criterion of significance.

## Results

### Patterns of albumin uptake around intercostal branch ostia changed with age

**Fig 1:**
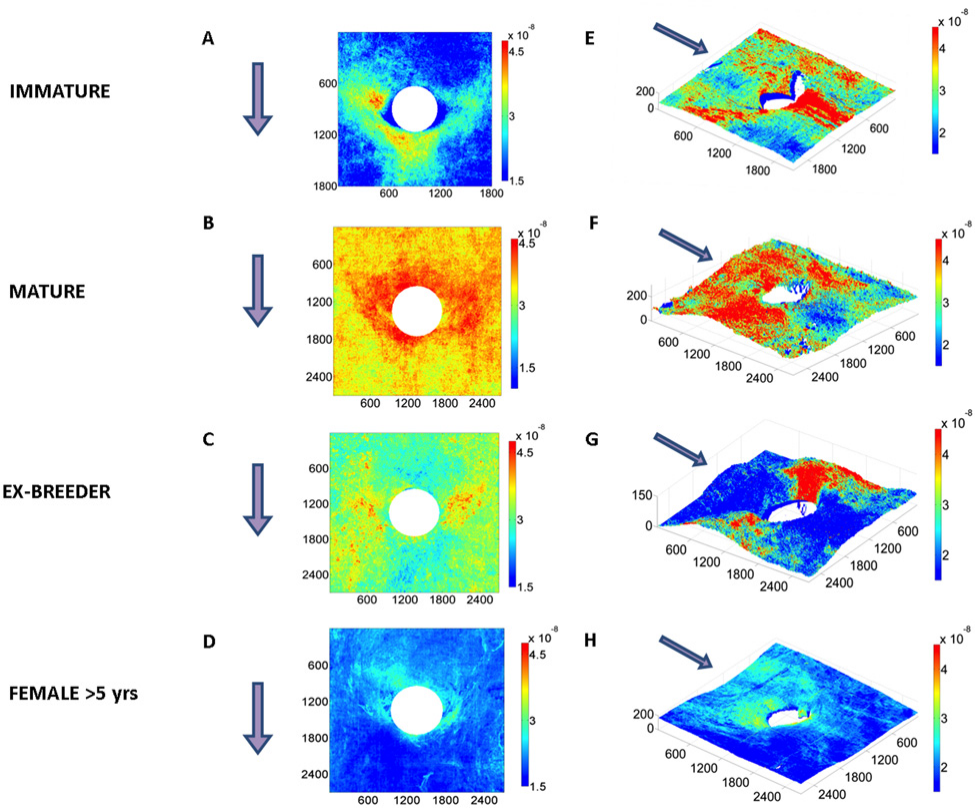
Maps of tracer uptake around branch ostia. Uptake by the aortic wall around intercostal branch ostia is shown for male rabbits aged (a) 9 weeks (immature), (b) 6 months (mature) and (c) 16–22 months (ex-breeder). Each map represents the average of at least 18 branches from at least 4 animals. (d) The equivalent pattern for 8 branches from 2 ex-breeder female rabbits aged >5 years. White circles indicate branch mouths. (e-h) An example at each age of a map of tracer uptake around a branch mouth superimposed on a 3D reconstruction of the corresponding aortic surface. Dimensions are shown in microns. Colour bars indicate mass transfer coefficients in cm/s. Arrows show the direction of mean aortic blood flow.

Fig. 1a-c show patterns of mass transfer coefficients around intercostal branch ostia in male rabbits aged 9 weeks (immature), 6 months (mature) and 16–22 months (ex-breeder). Each map represents the average of at least 18 branches from at least 4 animals. Fig. 1d shows the equivalent pattern for 8 branches from 2 ex-breeder female rabbits aged >5 years; it was not possible to obtain males or even a large number of females at this age, so these results must be regarded as exploratory.

Striking changes with age are apparent in Fig. 1. In the immature animals (Fig. 1a), uptake was highest in a triangular region downstream of the flow divider lip, extending to two patches at the lateral margins of the ostium. In the 16–22 month ex-breeder animals (Fig. 1c), there were again two patches of high uptake at the lateral margins but no patch downstream of the branch; indeed, apart from the flow divider lip itself, the downstream region appeared to have a particularly low uptake. The two lateral patches were connected to axial streaks located further from the ostium. At the intermediate age of 6 months (Fig. 1b), an intermediate pattern of mass transfer coefficients was seen: the lateral patches of high uptake were present but the reduction in uptake downstream of the branch was less marked. The data for the two 5-year-old female rabbits suggest that at even greater ages the lateral patches are smaller, the axial streaks are less marked, and uptake becomes relatively higher upstream of the branch.

**Fig 2:**
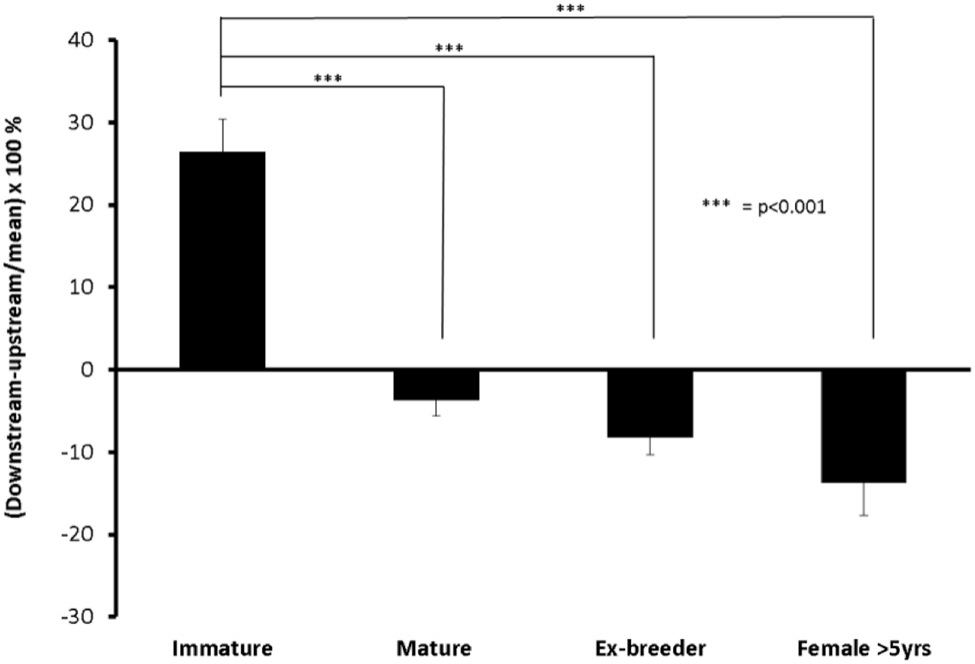
The difference in tracer uptake between regions upstream and downstream of the branch. Differences are expressed as a percentage of the mean of both regions at the different ages. Positive values indicate higher uptake downstream whilst negative values indicate higher uptake upstream. The trend with age between the three mature groups was significant (p<0.01). Data are shown as mean±SEM.

Fig. 2 quantifies these patterns by examining the difference in mass transfer coefficient between ROIs upstream and downstream of the branch, employing a metric used extensively in our earlier studies: in order to reduce the pattern of uptake to a single number, the upstream value is subtracted from the downstream value and the difference expressed as a percentage of the mean of both regions. Hence a positive number indicates that coefficients are greater downstream while a negative number indicates that they are greater upstream. A positive number was obtained for the immature animals and a negative one for all three adult groups (p<0.001 for the immature group vs each mature group), consistent with our previous data obtained by digital imaging fluorescence microscopy of longitudinal histological sections through the branch centerline [11, 12]. Furthermore, the data for 16–22 month male ex-breeder rabbits were on average >100% more negative than those for the 6-month-old rabbits, and the data for the females >5 years old were almost 100% greater than those for the ex-breeders, giving a continuous trend with age that was statistically significant (p<0.01).

**Fig 3:**
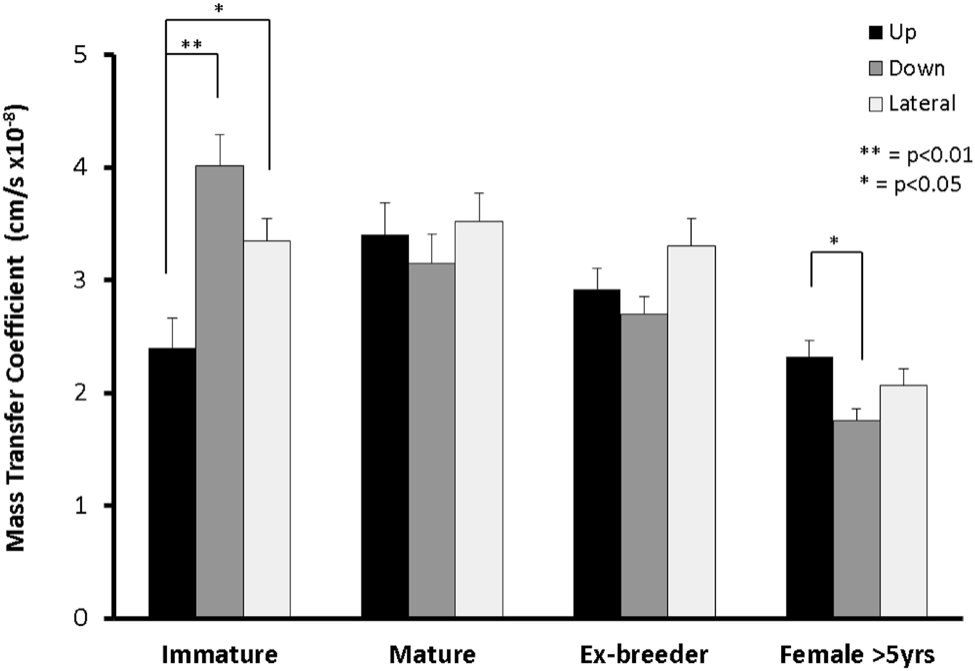
Mass transfer coefficients for the upstream, downstream and combined lateral regions of interest at different ages. Data are shown as mean±SEM.

Fig. 3 shows the mass transfer coefficients for the upstream, lateral and downstream ROIs separately. In immature rabbits the highest coefficients were obtained downstream of the branch, in mature rabbits the coefficients had increased upstream and decreased downstream giving a relatively uniform pattern, in ex-breeder rabbits the highest coefficients were lateral to the branch, and in rabbits >5 years old the highest coefficients were upstream. Statistical tests showed that uptake was significantly greater downstream of the branch than upstream in the immature rabbits (p = 0.0024); there were no such differences for the mature and ex-breeder rabbits (p = 0.68 and p = 0.31 respectively), whereas in the >5 year old female rabbits there was a significant trend in the opposite direction (p = 0.046). We attribute the latter difference to age rather than sex because three female rabbits aged 18 months (±1 week) did not give a more upstream pattern than the ex-breeder male rabbits of similar age shown in Fig. 1c—indeed all three gave a downstream pattern (data not shown). The difference between downstream and lateral regions approached significance (p = 0.12) in ex-breeders but not in the 6-month old rabbits (p = 0.60). Taken together, these results provide further statistical evidence for a continuing change in pattern with age.

In Fig. 1a-d, overall uptake appears highest in mature rabbits and lowest in the females >5 years. To quantify this result, Table 1 lists the mass transfer coefficients obtained for the whole square at each age group. All values lay within the range 2.03–3.32 x 10^-8^ cm/s. (The values may be slightly overestimated because autofluorescence, which is generally low compared to tracer fluorescence, was not subtracted from the measured intensities—see below). Consistent with the impression given by Fig. 1a-d, the whole-ostium figure was highest for the mature rabbits and lowest for those >5 years old, but the differences were not large: the only statistically significant difference was that between the ex-breeder and >5 year groups (p = 0.004).

**Table 1:** Effect of age and high-dose heparin on mass transfer coefficients around intercostal branch ostia.

Age Group	Control	High-dose heparin
Immature	2.86±0.14	2.24±0.28
Mature	3.32±0.26	3.61±0.35
Ex-breeder	3.10±0.18	3.74±0.27
Females >5 yr	2.03±0.09	

Units are cm/s x 10^-8^; mean±sem

Fig. 1e-h show an example at each age of a map of mass transfer coefficients superimposed on a 3D reconstruction of the corresponding aortic surface. Although some distortion of the 3D structure may have been introduced by the specimen-mounting procedure, robust features are still visible. In particular, it is apparent that in the immature branch (Fig. 1e) the lateral patches of high uptake occurred primarily downstream of, or lateral to, the margins of the flow divider whereas the lateral patches of high mass transfer coefficients seen in the >5-year example are upstream of or inside the flow divider margins (Fig. 1h). The 6-month animal seems to combine both distributions (Fig. 1f). It is also apparent, for example at 12–24 months (Fig. 1g), that high uptake often occurs in regions where the aortic wall merges with the wall of the side branch.

### Levels of rhodamine albumin varied with depth within the wall

**Fig 4:**
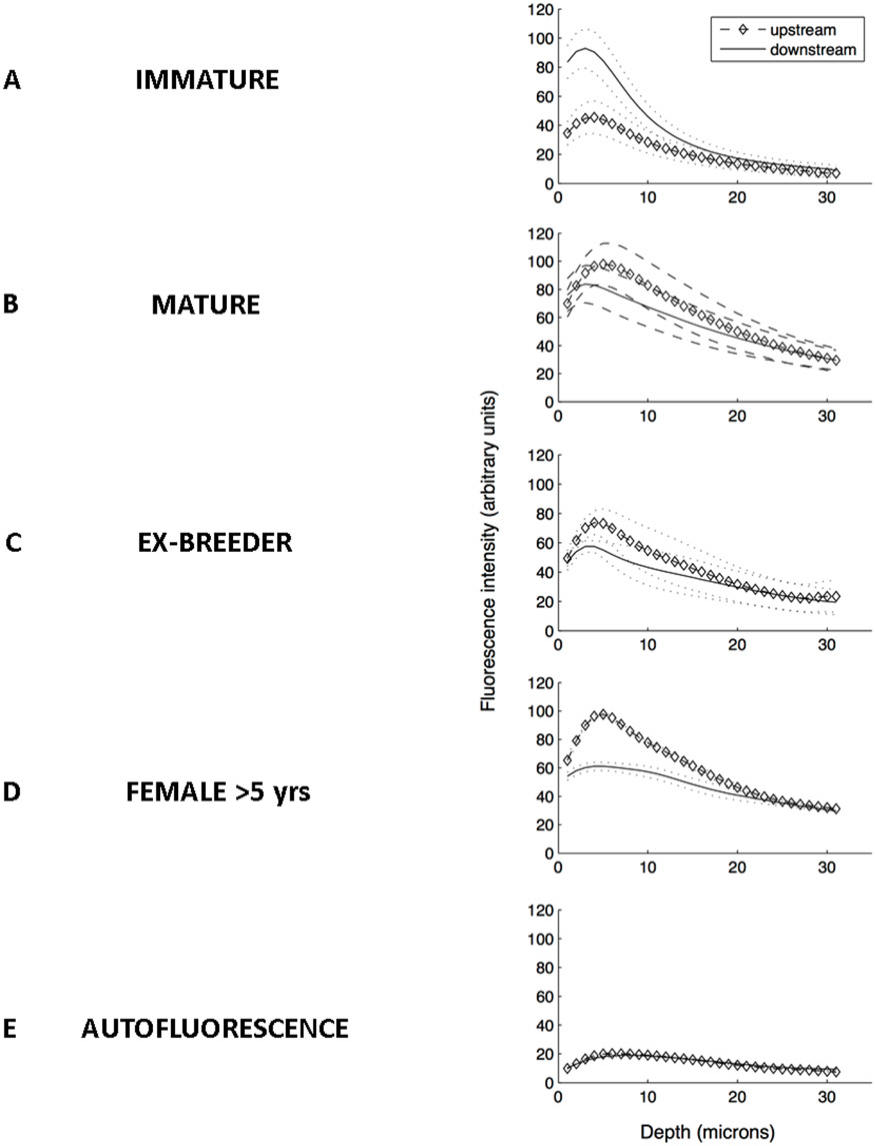
Variation in fluorescence intensity with depth into the wall. (a-d) Profiles of measured fluorescence intensity versus depth for regions of interest upstream and downstream of the ostium, averaged across all branches within each age group. (e) An equivalent profile for an animal not administered tracer, adjusted to account for different gain settings and hence indicating the comparable intensity of autofluorescence.

Fig. 4a-d show profiles of fluorescence intensity versus depth into the wall for ROIs upstream and downstream of the ostium, averaged across all branches within each age group. Fig. 4e shows an equivalent profile for an animal not administered tracer, adjusted to account for different gain settings and hence indicating the comparable intensity of autofluorescence. In all graphs the peak intensity appears to occur 3–7 μm into the wall but this may be an overestimate; the measured axial resolution of the objective lens is ~1.7 μm, leading to errors in the localisation of the luminal surface and some dispersion of the peak in both directions. Additionally, the drop-off with depth may be accentuated by scattering and absorption of light within the tissue. This effect was calibrated in our pilot study [2], which showed that measured values are attenuated by ~20% at 10 μm and by ~50% at 20 μm.

A number of consistent features are apparent. First, the pattern of peak heights corresponds to the pattern of mass transfer coefficients described above: peak intensities were greatest downstream of the branch in the immature animals and upstream in the three adult groups, and the relative difference between upstream and downstream peak heights increased from mature to ex-breeder to >5-year-old rabbits. Second, in immature rabbits most of the intensity attributable to tracer (which can be estimated by comparing the experimental curves with the autofluorescence curve) was located within the first 15–20 μm of the wall, but for the other age groups there appeared to be significant tracer penetration even to 30 microns. Third, at all ages the peak intensity was located slightly deeper in the wall for the upstream region than the downstream region in rabbits administered tracer.

### Patterns of uptake were similar at left and right branches

**Fig 5:**
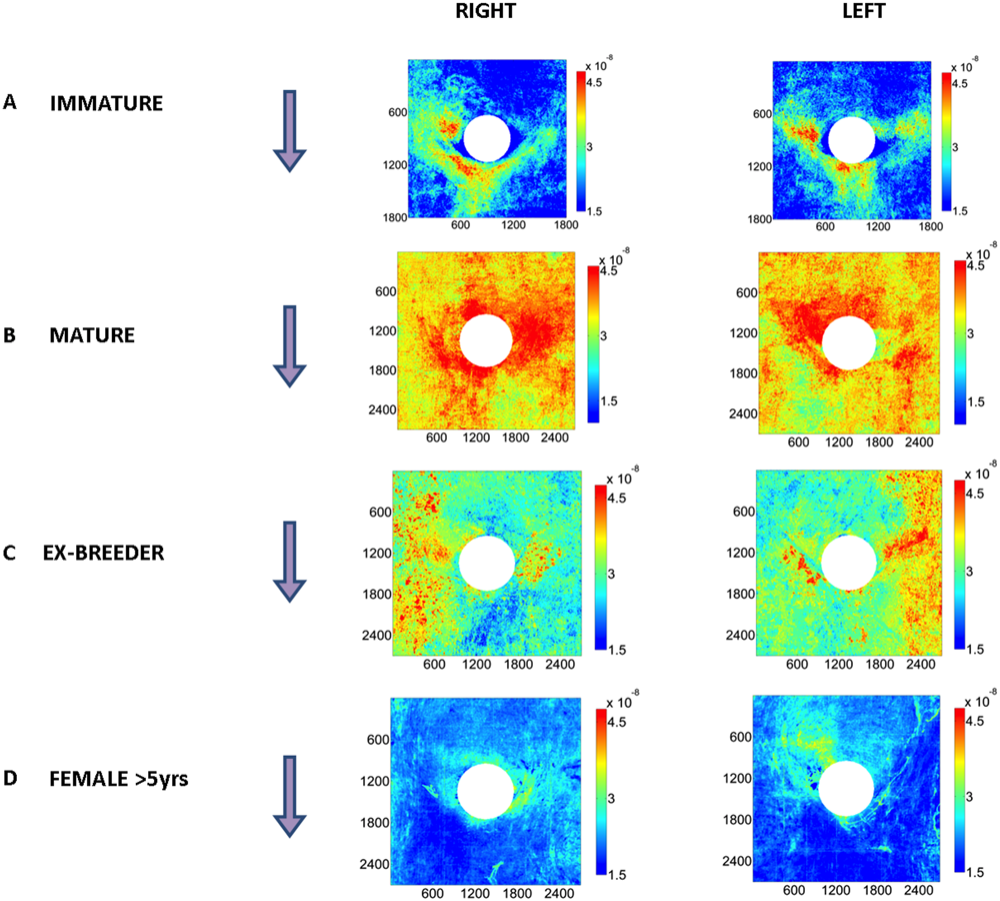
Uptake around left and right intercostal branch ostia. Maps of tracer uptake by the aortic wall around intercostal branch ostia in male rabbits aged (a) 9 weeks (immature), (b) 6 months (mature) and (c) 16–22 months (ex-breeder), and (d) female rabbits aged >5 years, displayed as in Fig. 1 except that branches have been subdivided according to whether they lie on the anatomical left or right side of the aorta.

Fig. 5a-d show average maps of mass transfer coefficients around branches for the 4 age groups as in Fig. 1 except that branches have been subdivided according to whether they lie on the anatomical left or right side of the aorta. For the immature rabbits, the patterns on the left-hand and right-hand sides were fundamentally similar. There was a comparable asymmetry on both sides of the aorta, with the lateral patch of high uptake being slightly greater on the right than the left margin of the branch mouth in both cases. A disparity between the left and right branches at this age was that the patch of highest tracer uptake downstream of the branch was skewed towards the anatomical right in the right-hand branches and to the left in the left-hand branches, but the difference was small. In the groups aged 6 months and 16–22 months, there was again a strong similarity between the patterns on the left and right sides, although in the latter group, away from the immediate vicinity of the branch mouth, there was a tendency for the axial streak of high uptake to be more pronounced on the side nearest to the lateral wall of the aorta (i.e. towards the left lateral wall for branches on the left side, and towards the right lateral wall for branches on the right side). In the female rabbits >5 years old, the patch of highest uptake was skewed towards the centreline—that is, towards the left in the right-hand branches and towards the right in the left-hand branches. However, the difference was slight and there was once more a strong similarity between the patterns on the two sides of the aorta.

The asymmetric trend described for the female rabbits >5 years old is visible in histograms showing mass transfer coefficients in the left and right lateral ROIs (S2 Fig.), but the visual impression that the skewing was slight is supported by statistical tests showing that coefficients did not differ significantly between these two regions in either left-hand or right-hand branches in any age group (0.1879≤p≤0.9483).

### Streaks of elevated mass transfer coefficients were seen in maps of larger areas

**Fig 6:**
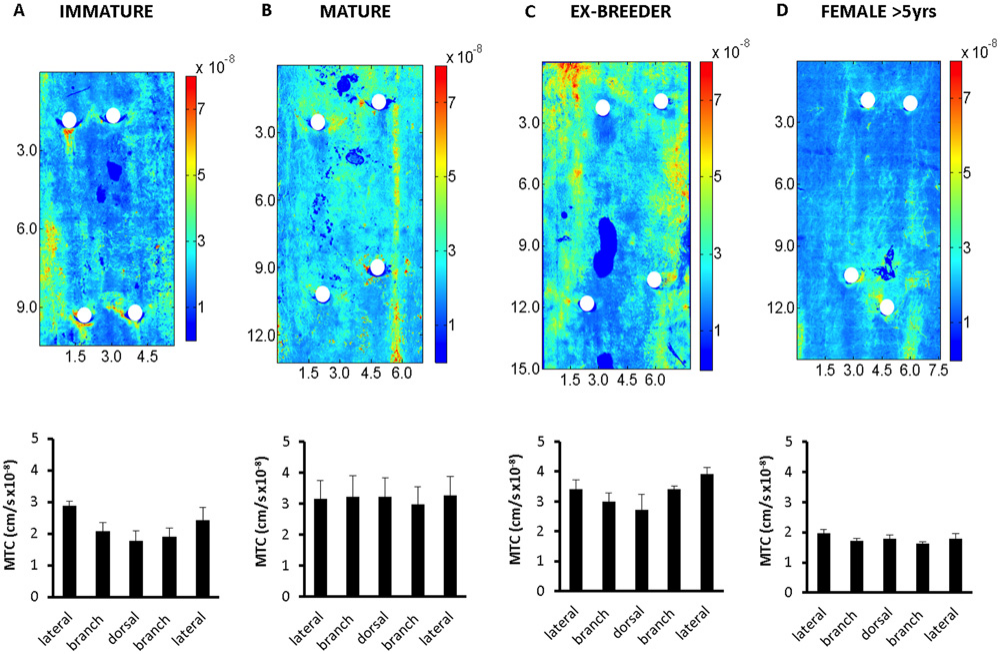
Uptake over wider areas. (a-d) Maps of mass transfer coefficients over a segment of aorta containing two pairs of intercostal branch ostia from a representative rabbit in each age group. Dimensions are shown in mm. Colour bars indicate mass transfer coefficients in cm/s. Mean aortic flow is from the top to the bottom of each map. Branch ostia are indicated by white circles. (Other holes are imaging artefacts). The histogram under each map shows the circumferential variation in mass transfer coefficients, quantified by averaging data in 5 elongated regions of interest located close to the left lateral margin and the right lateral margin of the map, between left branch ostia and between right branch ostia, and along the dorsal midline. (These regions avoided the areas immediately around the branches themselves, and the narrow longitudinal streaks of high uptake shown in subsequent figures.) The data (shown as mean±SEM) were obtained for all rabbits in each age group, not just the example maps.

Fig. 6a-d show maps of mass transfer coefficients for a segment of aorta containing two pairs of intercostal branch ostia in a representative rabbit from each age group. (Average maps were not created since the variation in ostial location between specimens would then require either misalignment of branch points or distortion of geometry.) The maps do not show the full circumference of the aorta but extend from its left to its right side. Consistent with Fig. 5, the fundamental features in the mean branch maps at each age (Fig. 1a-d) are visible for the left and right branches.

At all ages, longitudinal streaks of high uptake were seen in the lateral wall of the aorta of some rabbits; sometimes only one wall was affected and sometimes both were. The streaks were of two types—narrow and of very high intensity (e.g. Fig. 6b; this type is discussed in more detail below) and broader with lower intensity, although still brighter than the neighbouring tissue (Fig. 6a and 6c). In some animals there were also regions of elevated uptake near the dorsal midline (e.g. Fig. 6b), but these were rarer, shorter and less pronounced than the lateral streaks, and only the broader, lower intensity type was seen at this location.

Circumferential variation in mass transfer coefficients is quantified in the histogram under each map in Fig. 6. (The data were derived from all maps in each age group, not just the map shown as an example). None of the differences between individual locations reached significance.

### Narrow streaks of high uptake were dependent on fixation technique

**Fig 7:**
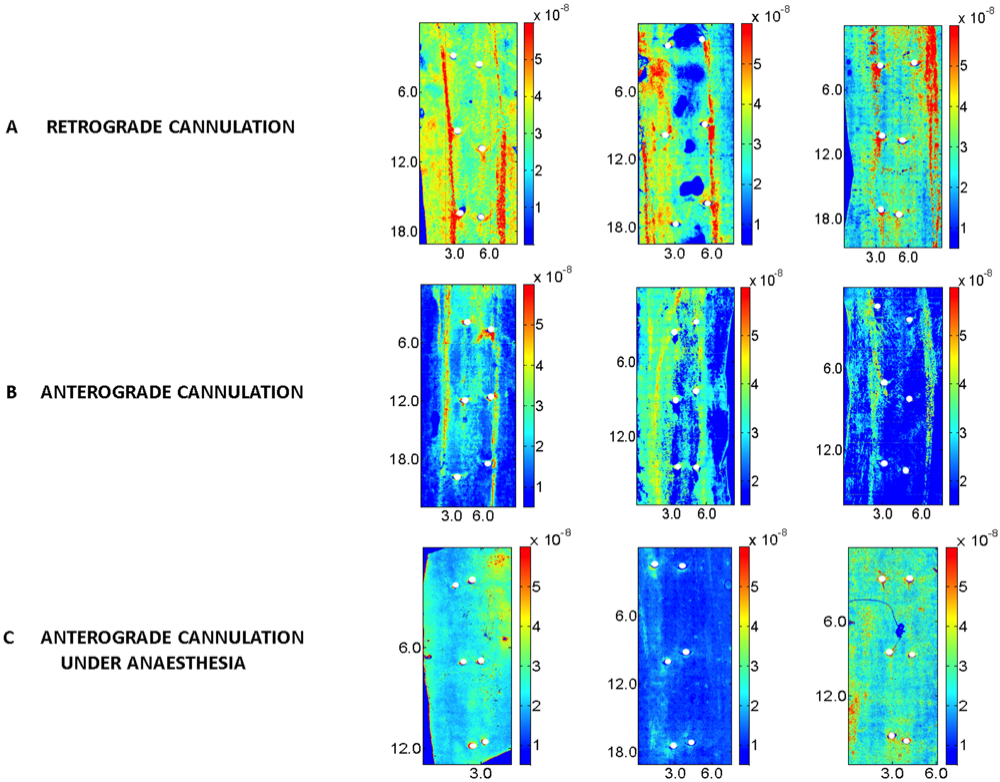
Effect of cannulation technique on streaks. Maps of mass transfer coefficients over a segment of aorta, displayed as in Fig. 6. (a) Three aortas flushed and fixed *via* a retrograde cannula inserted after death. (b) Three aortas flushed and fixed via an anterograde cannula inserted after death. (c) Three aortas flushed and fixed via an anterograde cannula inserted under anaesthesia.

The narrow streaks of high uptake observed lateral to branch ostia and extending along much of the length of the scan (e.g. Fig. 6b) occurred most often in adult animals but were also present in over half of the young scans. In cases where two streaks were present, they were approximately parallel and arranged either side of the branch pairs. Three further examples are shown in Fig. 7a. They were also seen in 5 out of 6 vessels fixed *via* an anterograde cannula inserted into the proximal descending aorta after death (e.g. Fig. 7b) but no comparable streaks were seen in 8 vessels fixed *via* an anterograde cannula inserted under anaesthesia, to prevent prolonged exposure of the vessel to tracer in the absence of blood pressure (e.g. Fig. 7c). The broader, lower intensity lateral streaks were still apparent in the latter.

### Narrow streaks of high uptake were still apparent when in vivo exposure to tracer was minimised

**Fig 8:**
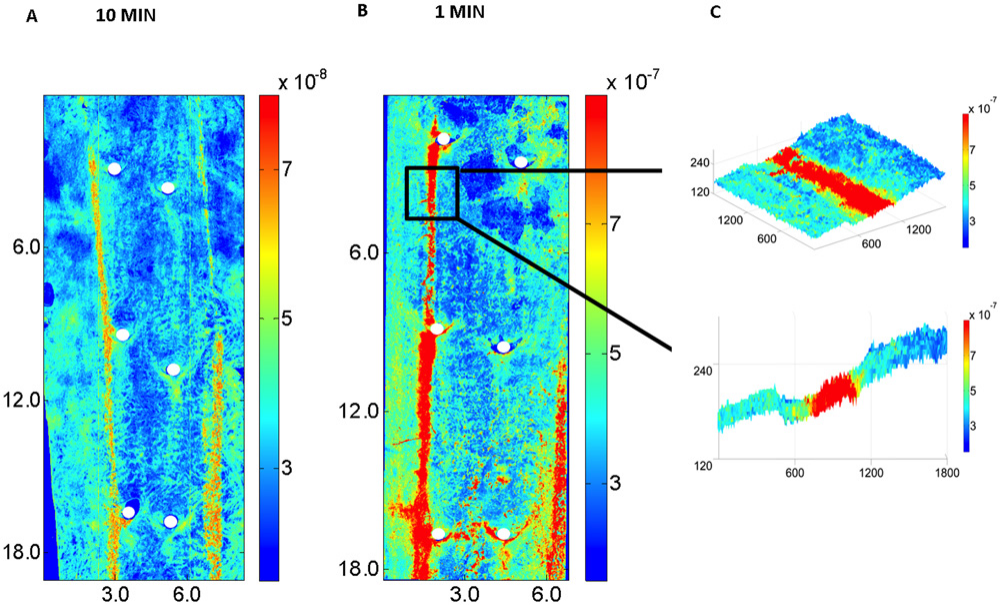
Effect of duration of uptake on streaks. (a and b) Maps of mass transfer coefficients over a segment of aorta, displayed as in Fig. 6. The aortas were exposed to circulating tracer for (a) 10 or (b) 1 min *in vivo* and then flushed and fixed *via* a retrograde cannula inserted after death. (c) A portion of map (b) superimposed on a 3D representation of wall structure, and a 2D slice through the 3D image. Note that depth into the wall is shown on an expanded scale compared to the axial and circumferential dimensions (all in microns).

Tracer was administered 1 min rather than 10 min prior to euthanasia in some animals in order to examine the effect on the narrow streaks of drastically reducing the time the vessel was exposed to tracer under physiological conditions whilst not affecting the duration of exposure to tracer between death and flushing the vessel. (Flushing and fixation were performed via a retrograde cannula in the distal thoracic aorta, as usual). Fig. 8a and 8b show maps of mass transfer coefficients after 10 and 1 min exposure, respectively. Fig. 8c shows a portion of the map for a 1-minute experiment superimposed on a 3D representation of wall structure and a 2D slice through the 3D image. The narrow streak is relatively more accentuated with only 1 min of *in vivo* exposure, and it is also clear that the streak lies within a groove (width c. 750 μm, depth c. 30 μm) where the aortic surface is disrupted in some way.

Because the narrow lateral streaks appear artefactual, Figs. 1a-d, 3 and 5a-d were recomputed after omitting branches affected by these features. The resulting averages (S3, S4 and S5 Figs., respectively) are virtually indistinguishable from the original Figures.

### Heparin affected patterns of uptake

**Fig 9:**
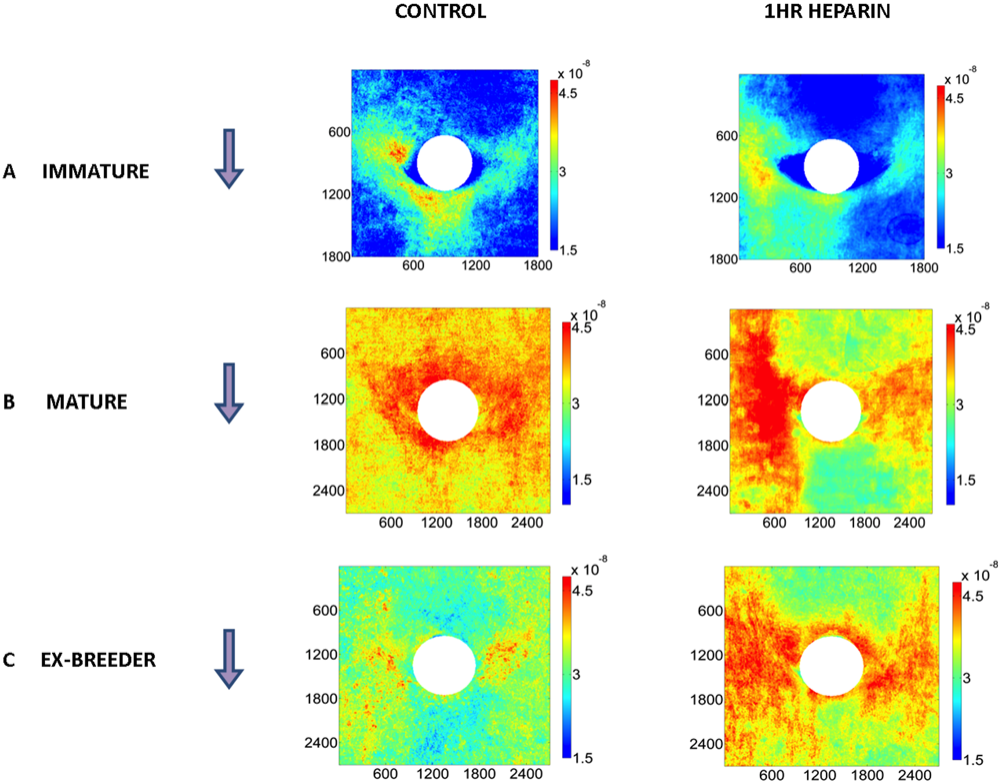
Effect of high-dose heparin on uptake patterns. Maps of uptake around branches of male rabbits aged (a) 9 weeks, (b) 6 months and (c) 16–22 month rabbits after 1h exposure to high-dose heparin, displayed as in Fig. 1. (Maps show means of 22 branches from 4 immature rabbits; 22 branches from 4 mature rabbits; and 18 branches from 3 ex-breeders) The equivalent control patterns from rabbits receiving low-dose heparin for 2 min are repeated from Fig. 1 to facilitate comparison.

**Fig 10:**
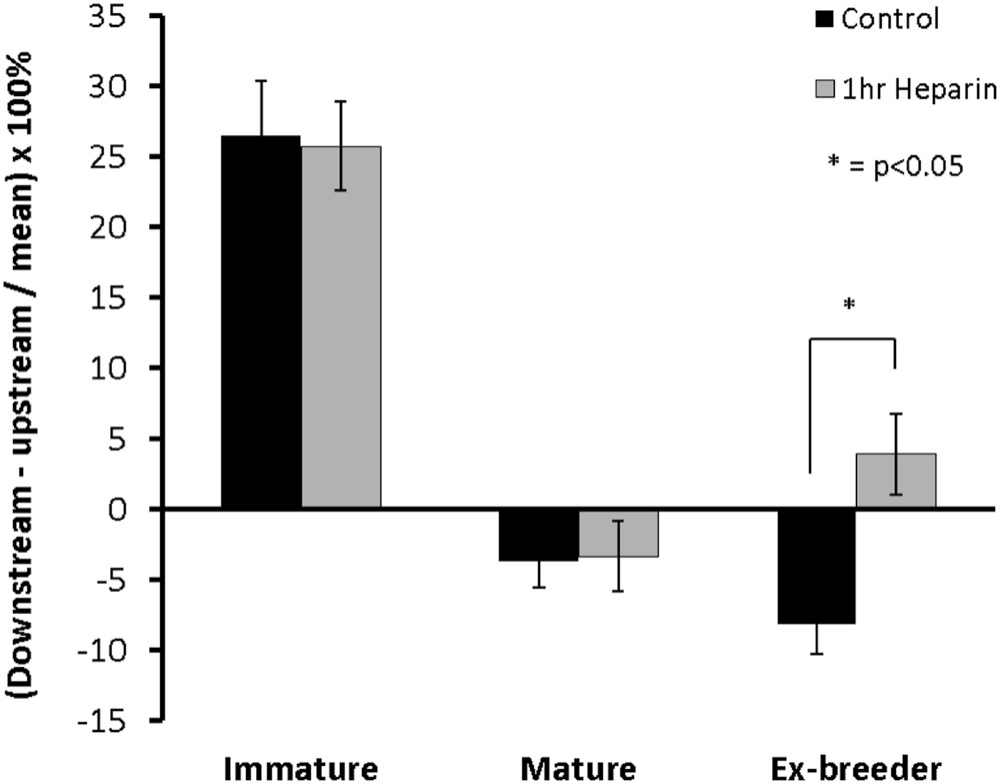
Effect of high-dose heparin on uptake upstream and downstream of the branch. Effect of pre-treatment with high-dose heparin for 1h on the difference in transport between regions of interest upstream and downstream of the branch in rabbits of different age, quantifying aspects of the patterns shown in Fig. 9. Data are presented as described in Fig. 2. The direction of the difference was reversed by 1h heparin treatment in ex-breeder animals (p = 0.0131).

Fig. 9a-c show the mean patterns of uptake around branches in rabbits aged 9 weeks, 6 months and 16–22 months after prolonged exposure to high-dose heparin. The equivalent control patterns from Fig. 1 are repeated alongside to facilitate comparison. There were no significant effects of heparin on mass transfer coefficients computed for the whole maps (Table 1). Fundamental features of the maps of mass transfer coefficients were also unchanged at all ages, but a number of minor changes were apparent: skewing of the downstream triangle in immature animals, accentuated lateral streaks at 6 months and increased transport impinging on the region downstream of the branch at 16–22 months. The latter trend had a strong, significant effect on the difference in transport between ROIs upstream and downstream of the branch (Fig. 10), which changed from upstream>downstream in controls to downstream>upstream after high dose heparin (p = 0.0131).

## Discussion

Uptake of plasma macromolecules by the arterial wall is less widely studied than other critical factors in atherogenesis and that may in part reflect a lack of convenient techniques. We recently presented a practicable method and a pilot study that demonstrated its accurate quantification, high throughput and high resolution over a wide area [2]. The technique was further developed and exploited in the present study in order to address questions concerning the predilection of atherosclerosis for sites in the vicinity of arterial branch points.

Improvement in the automated identification of the luminal surface of vessels viewed *en face* gave better rejection of glare from the surface and hence better definition of the patterns of mass transport. This was particularly apparent in mature aortas, which have a more subtle pattern around branch points than immature aortas: although the pilot study found a rather uniform level of fluorescent tracer at these locations, the present study demonstrated a distinctive distribution. Even in the immature animals, the present investigation gave a more pronounced pattern than the pilot study, with a substantially larger difference between regions upstream and downstream of the branch.

Using the 3D capabilities of the technique to create profiles of fluorescence intensity versus depth into the wall showed that peak tracer concentrations occur within a few microns of the luminal surface and that tracer concentration falls rapidly with distance into the wall. The peak heights of the profiles showed the same trend as uptake integrated over the first 10 μm: they were greater downstream of the branch than upstream in immature animals but showed the opposite pattern in adults, with the relative difference between regions increasing in each successive age group. (Note that the age at which the pattern reverses depends on the strain of rabbit [13].) Differences in the shape of the profiles obtained in upstream and downstream regions may reflect differences in endothelial properties or in the structure of the underlying wall.

The 3D capabilities were also used to project maps of mass transfer coefficients on to reconstructions of the luminal surface. Examples obtained using this method demonstrated for the first time that although patches of high uptake are seen at the sides of the branch in both immature and aged rabbits, these patches can occur on different sides of the flow divider lip, and may therefore reflect exposure of the wall to quite different mechanical stresses.

The quantitative nature of the method was used to produce maps of absolute mass transfer coefficients, permitting comparison with other methods. Mean coefficients lay in the range 1.7–4.1 x 10^-8^ cm/s, which is consistent with previous data obtained using different techniques (e.g. [14]). The good agreement suggests that the calibration procedures used in the present method and the inherently linear relation between fluorescence intensity and concentration lead to an accurate assessment of tracer uptake by the wall.

Uptake around aortic side branches has been examined in immature rabbits in many earlier studies. Consistent with previous data for macromolecular tracers of varying size—albumin [15], horseradish peroxidase [16], and low density lipoprotein (LDL) [5, 17]—we demonstrated a pronounced triangle of high uptake downstream of the ostium. Lesion prevalence shows a similar distribution in the immature rabbit aorta [18] and in human fetuses, newborns and infants [19]. Patterns of uptake have been mapped around side branches in mature rabbits only twice, once in our pilot study [2] (which, as already discussed, showed nearly uniform uptake) and once by using a laborious serial sectioning technique on a small sample [15]. The present study adds to that small data set and is the first to investigate patterns in adult animals of different ages. Not only did the percentage difference between upstream and downstream areas increase significantly with age in the adult groups, but there appeared to be qualitative differences in the pattern as well: there were longitudinal stripes of high uptake lateral to the branch at 6 months and 16–22 months, giving a pattern resembling that reported for lesions in mature cholesterol-fed rabbits [20] and young adult humans [3], whilst at greater ages there was a more upstream pattern, similar to that found for lesions in older people [3]. Although patterns of transport have not been measured around human arterial branch points, these correlations support the supposition that lesion prevalence is determined by transport variations in people as well as in rabbits.

We also investigated whether patterns of uptake around branches are caused by the branches themselves or reflect larger-scale patterns within which the branches happen to be situated. The latter hypothesis was suggested by our computational simulations of aortic fluid dynamics, which show that large-scale patterns of shear stress in the descending thoracic segment sweep through branch points and modify aspects of the pattern of shear around them [4]. Since shear and wall transport properties have been linked [21], it is plausible that a parallel phenomenon occurs for the latter. Consistent with the patterns being predominantly determined by the branches themselves, only minor differences were seen between ostia on the left and right sides of the aorta. Similarly, the essential features of the patterns were seen in left- and right-hand branches when uptake was mapped in larger segments of the proximal descending thoracic aorta. Variation in uptake around the circumference of the aorta was complex and requires further investigation, but the longitudinal stripes of high uptake often seen towards the lateral walls of the vessel seem to have had little influence on transport around the branches themselves.

A novel finding in maps of the larger aortic segments was the presence of high-uptake streaks of two different widths. Our computational studies of rabbit aortic flow have demonstrated longitudinal features in maps of various wall shear stress metrics [4, 22, 23], but these are much wider than the narrower type of streak of high uptake observed in the present study. Superficially, that seemed like evidence for a discrepancy between patterns of uptake and patterns of shear. Further investigation resolved this discrepancy by showing that the narrower streaks are artefacts arising from *post mortem* tracer uptake occurring in arteries damaged by depressurization: unlike the broader streaks, they were absent in vessels cannulated under anaesthesia (and hence not depressurized until immediately before tracer was flushed from the lumen), and they were relatively accentuated in animals where tracer was injected just prior to death and in which any *post mortem* uptake would therefore constitute a larger fraction of the total. These findings are consistent with the idea that depressurization causes endothelial damage [6]. Artefactual streaks may have affected earlier studies. For example Herrmann *et al*. [5] assessed 10-minute uptake of radiolabelled LDL in immature rabbits; the aortas were fixed *via* a cannula inserted into the left ventricle *post mortem*. In agreement with the present study, streaks of high LDL uptake were observed in the thoracic aorta.

Pre-treatment with heparin has previously been found to accelerate the clearance of 70 kDa dextran from the circulation, a property that is likely to be dominated by microvascular rather than arterial permeability; hyaluronidase had a similar effect [7]. Heparin also reduced flow-mediated arteriolar dilatation and diminished the bioavailability of NO [7]. A plausible explanation for these phenomena is that heparin releases proteins bound to heparan sulphate in the endothelial glycocalyx, thereby interfering with the shear-dependent release of NO [7]. There is abundant evidence for influences of NO on permeability, but the nature of the effect varies with vessel and preparation. In our earlier studies, which examined uptake upstream and downstream of intercostal branch ostia, but only along their centerline, the difference between upstream and downstream regions was unaffected when NO synthesis was inhibited in immature rabbit aortas perfused *in situ* but was reversed in rabbits aged around 12 months [8]. The effect seen in the mature perfused vessels was replicated *in vivo*, but the magnitude of the reversal was smaller than *in vitro*, suggesting that other effects of NO in the intact animal (such as alteration of blood pressure) had a countervailing influence [9].

In the present study, pre-treatment with heparin had the same effects on differences between regions upstream and downstream of the branch as previously observed with NO inhibitors: the difference was unaffected in immature rabbits but was reversed in ex-breeders. Furthermore, the values obtained in the ex-breeders with and without heparin pre-treatment were remarkably similar to those obtained in the study where NO was inhibited in rabbits of similar age *in vivo*. Given these similarities, and the evidence that heparin disrupts flow-mediated arteriolar dilatation and reduces the bioavailabity of NO, we assume in the following that the effects of heparin pre-treatment which we observed were caused by an interference with the NO pathway.

The effects of NO inhibition in our earlier work led us to suggest that there is a change in signaling pathways with age, the mature pattern being NO-dependent and the immature pattern not. It was assumed that inhibition of the NO pathway in mature animals caused a reversion to the immature one. In the present study, the new confocal technique allowed us to look at mass transport coefficients all around branches, rather than just upstream and downstream of them. The maps of transport (Fig. 8a-c) showed that the heparin-induced reversal in the difference between upstream and downstream regions in ex-breeders did not reflect a reversion to the immature transport pattern but instead resulted from a quite subtle change to the normal pattern. There were also subtle changes in the immature pattern—they just happened not to affect the difference between regions upstream and downstream of the branch.

If these results also apply to the effect of inhibiting NO production *in vivo*, two significant inferences can be drawn. First, the results would explain why chronic administration of NO inhibitors failed to produce a reversion to the junior pattern of diet-induced lesions around intercostal branch ostia in mature rabbits but *did* produce a small reversal in the difference between upstream and downstream regions along the branch centerline [9]. Second, the results would contradict our earlier conclusion that there is a change in mechanotransduction pathways with age [8], making it more likely that the change in transport pattern with age is a consequence of a change in the mechanical forces themselves. The existence of changes in mechanical forces is supported by studies of immature and mature rabbit aortas that mapped endothelial nuclear elongation, a shear-sensitive property, around intercostal branch ostia [24]; nuclei were most elongated downstream of branches in immature rabbits but were least elongated at this location in mature animals. The existence of changes in mechanical forces is also supported by computational fluid dynamics studies that mapped a novel metric, the transverse wall shear stress (transWSS), characterising multidirectional flow [23, 25]. TransWSS correlated better than conventional shear metrics with the age-related patterns of disease [25].

## Supporting Information

S1 Fig Selecting regions of interest. For this branch from a young animal, a square of 300 x 300 pixels (1800 x 1800 μm), centred on the ostium, was cropped from the map of aortic mass transfer coefficients. Within the square, regions of interest (shown in white) with dimensions 75 x 100 pixels (450 x 600 μm) were delineated upstream, downstream and at the sides of the ostium for further analysis.
(TIFF)

S2 Fig Asymmetry of mass transfer coefficients. (a-d) Mass transfer coefficients (MTC; mean±SEM) for the left and right lateral ROIs, shown separately for left and right intercostal branch mouths at the four different ages. Nested ANOVA found no significant differences between left and right ROIs in any panel (0.1879≤p≤0.9483).
(TIFF)

S3 Fig Maps of uptake around branches unaffected by narrow streaks of high uptake. Fig. 1a-d recomputed after omitting branches affected by the artefactual narrow streaks of high uptake.
(TIFF)

S4 Fig Mass transfer coefficients in regions of interest around branches unaffected by narrow streaks of high uptake Fig. 3 recomputed after omitting branches affected by the artefactual narrow streaks of high uptake. (TIFF)

S5 Fig Maps of uptake around left and right branches unaffected by streaks of high uptake. Fig. 5a-d recomputed after omitting branches affected by the artefactual narrow streaks of high uptake.
(TIFF)
