# Supplementary figures and images for: Mass Transport Properties of the Rabbit Aortic Wall
Source: PLoS One. 2015 Mar 17;10(3):e0120363. doi: 10.1371/journal.pone.0120363 (PMC4363731; doi:10.1371/journal.pone.0120363)

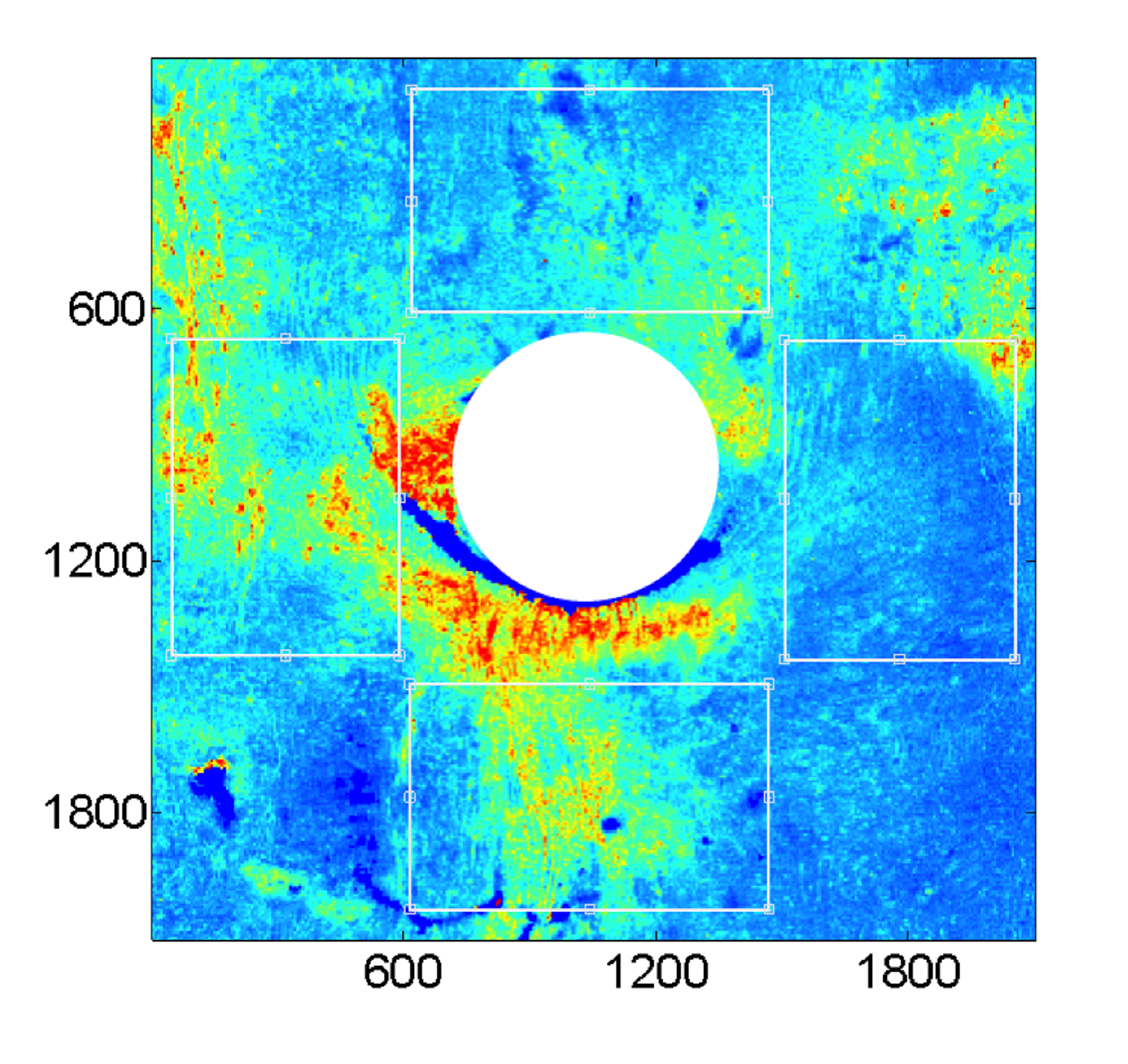

Supplement: S1 Fig — For this branch from a young animal, a square of 300 x 300 pixels (1800 x 1800 μm), centred on the ostium, was cropped from the map of aortic mass transfer coefficients. Within the square, regions of interest (shown in white) with dimensions 75 x 100 pixels (450 x 600 μm) were delineated upstream, downstream and at the sides of the ostium for further analysis. (TIFF) [file pone.0120363.s001.tiff]

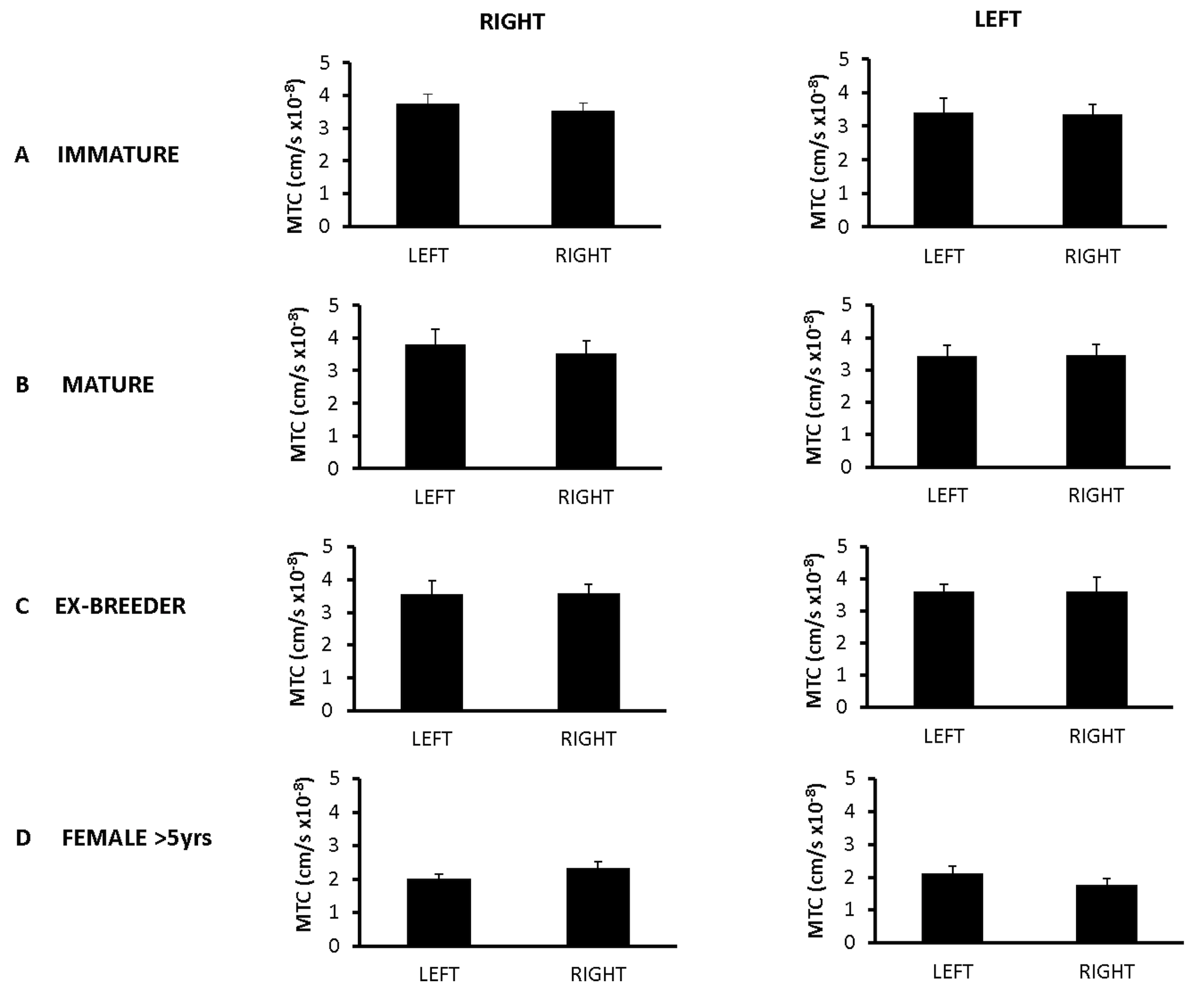

Supplement: S2 Fig — (a-d) Mass transfer coefficients (MTC; mean±SEM) for the left and right lateral ROIs, shown separately for left and right intercostal branch mouths at the four different ages. Nested ANOVA found no significant differences between left and right ROIs in any panel (0.1879≤p≤0.9483). (TIFF) [file pone.0120363.s002.tiff]

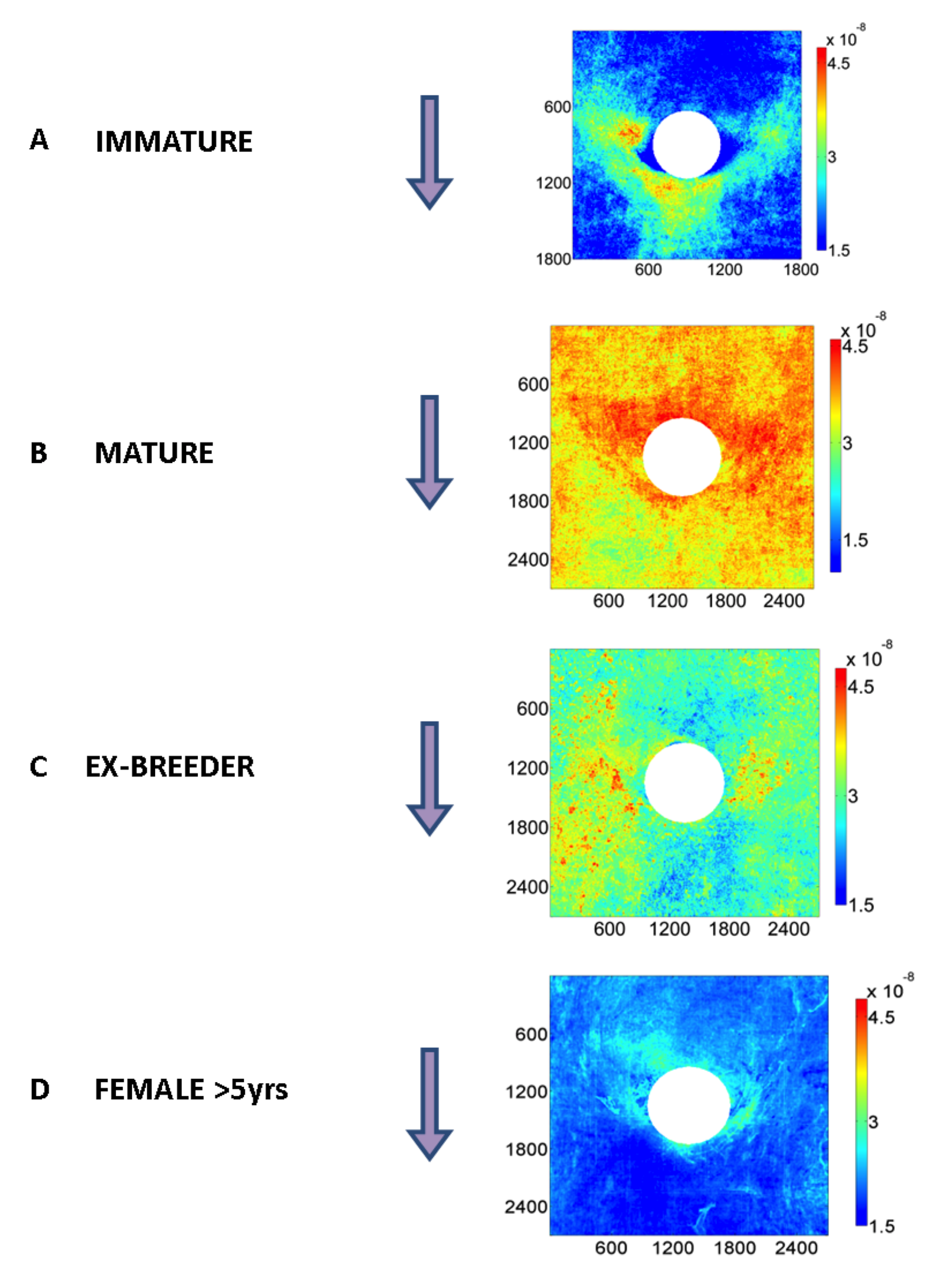

Supplement: S3 Fig — Fig. 1a-d recomputed after omitting branches affected by the artefactual narrow streaks of high uptake. (TIFF) [file pone.0120363.s003.tiff]

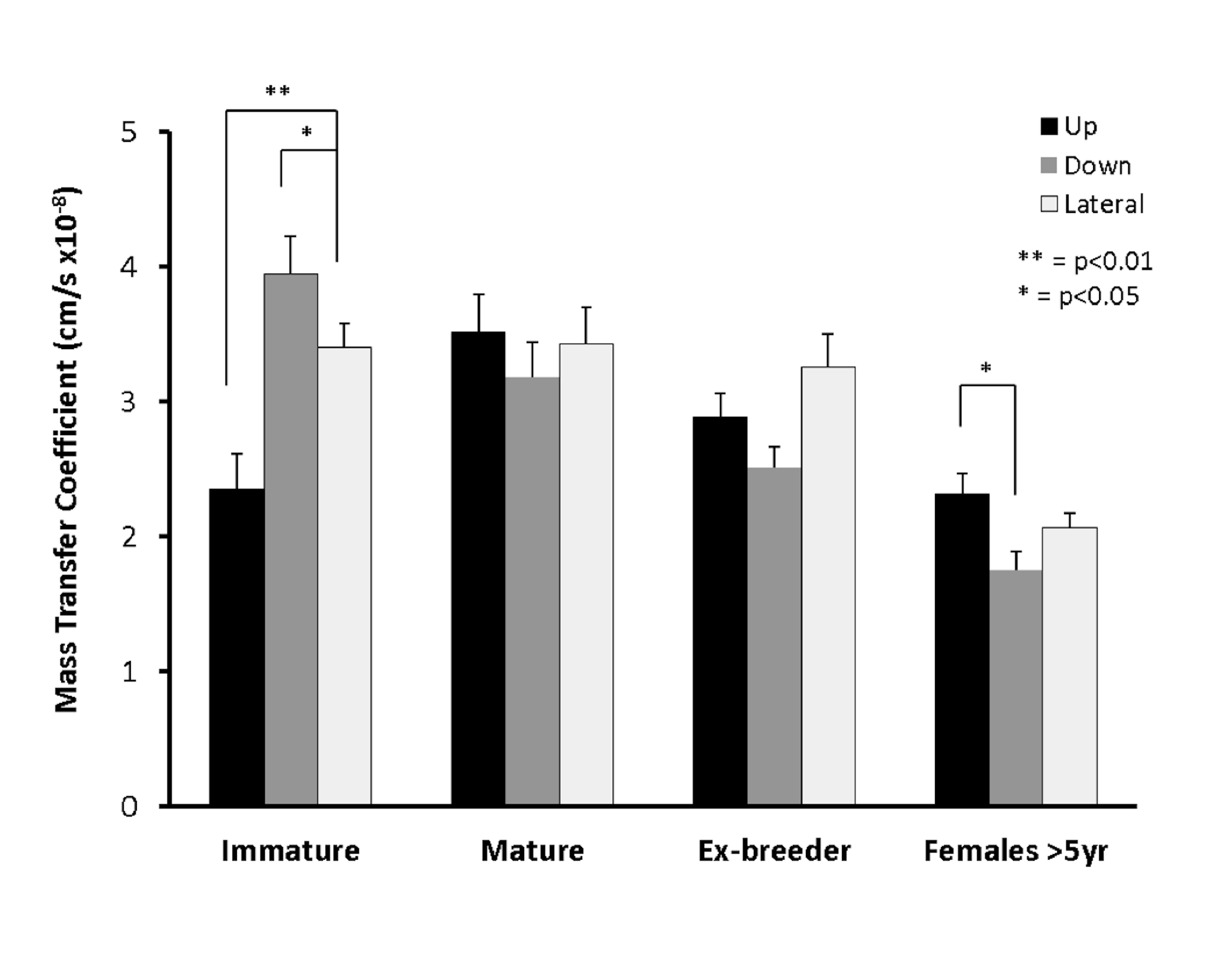

Supplement: S4 Fig — (TIFF) [file pone.0120363.s004.tiff]

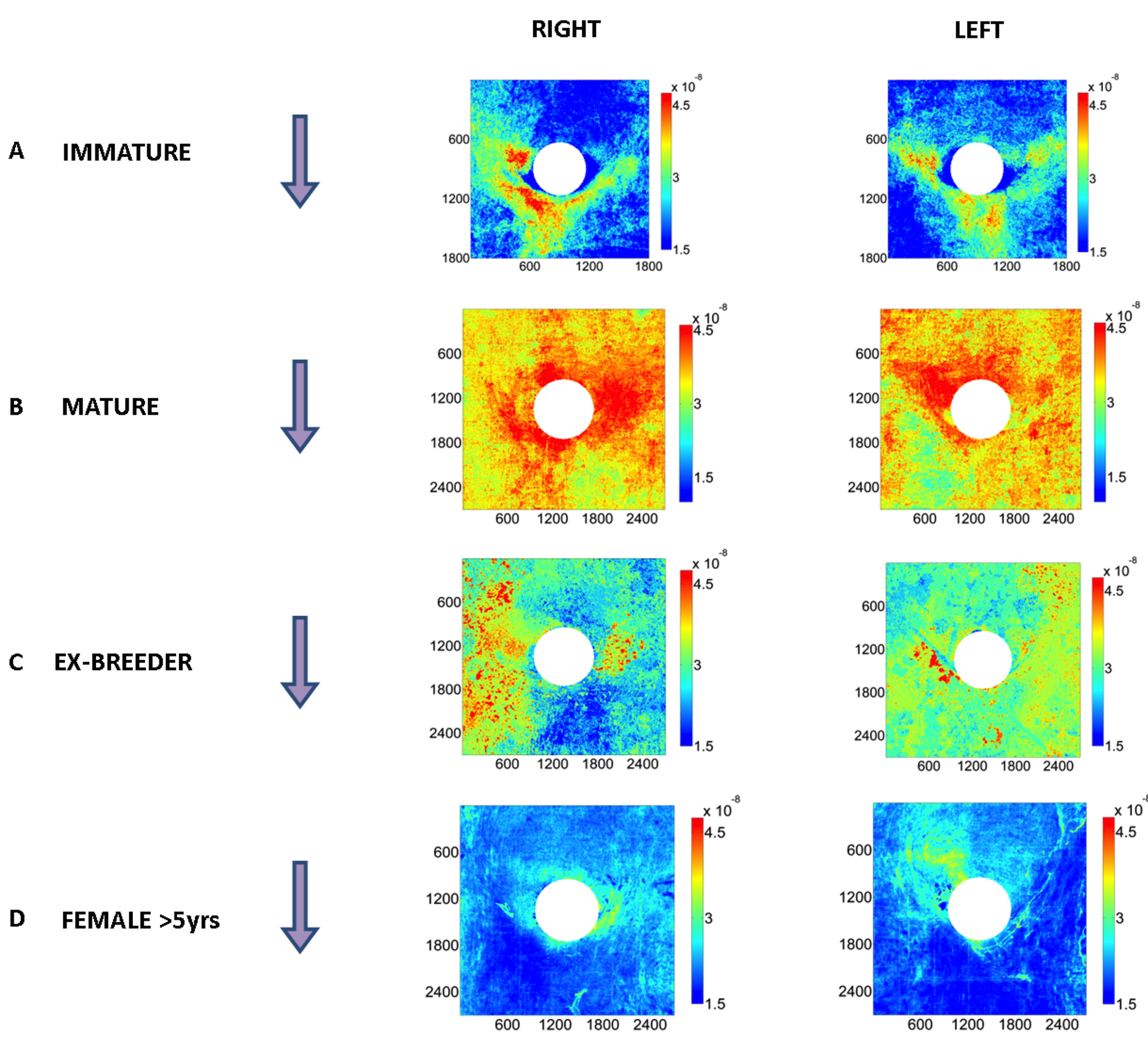

Supplement: S5 Fig — Fig. 5a-d recomputed after omitting branches affected by the artefactual narrow streaks of high uptake. (TIFF) [file pone.0120363.s005.tiff]
